# Numerical accuracy of closed-loop steady state in a zero-dimensional cardiovascular model

**DOI:** 10.1098/rsta.2024.0208

**Published:** 2025-04-02

**Authors:** Nick van Osta, Gitte van den Acker, Tim van Loon, Theo Arts, Tammo Delhaas, Joost Lumens

**Affiliations:** ^1^ Department of Biomedical Engineering, Cardiovascular Research Center Maastricht (CARIM), Maastricht University, Maastricht, The Netherlands

**Keywords:** computer model and simulation, human cardiovascular physiology, credibility assessment, CircAdapt framework

## Abstract

Closed-loop cardiovascular models are becoming vital tools in clinical settings, making their accuracy and reliability paramount. While these models rely heavily on steady-state simulations, accuracy because of steady-state convergence is often assumed negligible. Using a reduced-order cardiovascular model created with the CircAdapt framework as a case study, we investigated steady-state convergence behaviour across various integration methods and simulation protocols. To minimize the effect of numerical errors, we first quantified the numerical errors originating from integration methods and model assumptions. We subsequently investigate this steady-state convergence error under two distinct conditions: first without, and then with homeostatic pressure-flow control (PFC), providing a comprehensive assessment of the CircAdapt framework’s numerical stability and accuracy. Our results demonstrated that achieving a clinically accurate steady state required 7–15 heartbeats in simulations without regulatory mechanisms. When homeostatic control mechanisms were included to regulate mean arterial pressure and blood volume, more than twice the number of heartbeats was needed. By simulating a variable number of heartbeats tailored to each simulation’s characteristics, an efficient balance between computational cost and steady-state accuracy can be achieved. Understanding this balance is crucial as cardiovascular models progress towards clinical use.

This article is part of the theme issue ‘Uncertainty quantification for healthcare and biological systems (Part 2)’.

## 1. Introduction

Computational models of the cardiovascular system are increasingly used for (pre-)clinical research, education, healthcare and therapy development [1]. These models provide valuable insights into complex physiological processes, enabling a deeper understanding of cardiac function and disease mechanisms. As these models move closer to clinical applications, ensuring their numerical credibility becomes paramount.

One such model is the CircAdapt model, a reduced-order biophysical model designed to simulate the cardiovascular system dynamics across both healthy and pathophysiological conditions [2–5]. The biophysical basis of the model enables exploration of cause-and-effect relationships between tissue abnormalities, cardiac pump function and circulatory hemodynamics. Key elements are (i) the one-fibre model, relating wall stress to cavity pressure [6], (ii) the TriSeg module to simulate inter-ventricular interaction over the inter-ventricular septum [5] and (iii) the closed-loop circulation with a homeostatic pressure-flow control (PFC) [2].

While the CircAdapt model has been valuable for research, education and translational studies [7,8], the complexity of cardiovascular simulation demands rigorous verification. Recent guidelines [9,10] emphasize the need for structured approaches to verification and validation of the model used. Effective software quality assurance ensures that computational models perform as expected under various (patho-)physiological conditions [11]. For example, established platforms used for the simulation of cardiovascular electrophysiology such as OpenCarp [12], Chaste [13–15] and OpenCor [16] have implemented unit tests, comparing their simulation results against reference data to test the expected behaviour of the software [17].

Cardiovascular mechanics models typically involve more complex partial differential equations. To reduce the effect of initial conditions of these equations, multiple heartbeats are simulated to converge to a haemodynamic steady state. This steady state is not only a result of the parameterization, but also of the initial state variables, or a result of adaptation or regulation [2,3,17]. Reducing the number of simulated heartbeats can significantly decrease computation time but potentially introduce inaccuracies by failing to achieve a true steady state.

In this study, we examine how the number of simulated heartbeats affects steady-state accuracy in cardiovascular models, particularly in the CircAdapt framework. To enable precise quantification of the steady-state convergence error, we first quantify the numerical errors originating from integration methods and model assumptions. We subsequently investigate this steady-state convergence error under two distinct conditions: first without, and then with homeostatic PFC, providing a comprehensive assessment of the CircAdapt framework’s numerical stability and accuracy.

## 2. Methods

In this study, we use the CircAdapt framework—a modular toolbox to create fast, functional models of the cardiovascular system—to create a zero-dimensional lumped parameter model of the heart and circulation. Currently, it consists of two main components: the CircAdapt library, a C++ engine optimized for efficient computation and pyCircAdapt, a Python wrapper that facilitates easy interaction with the system. A key feature of the CircAdapt framework is its physiology-driven approach to module design, which applies fundamental principles of physics to simulate physiological processes accurately in a simple and efficient manner.

The basis of this physiology-driven approach to simulate cardiovascular function is the one-fibre model [6]. This model defines the relationship between cavity and wall volume, fibre strain and the ratio of fibre stress over cavity pressure by assuming a simplified thick-walled rotationally symmetric geometry and conservation of energy. Within the framework, the linearized one-fibre model is integrated into the Wall component with the Patch module providing the corresponding time- and stretch-dependent stress–strain relationship, as previously described [4]. The Chamber [2] and TriSeg [5] modules describe atrial and ventricular dynamics, respectively, with the Wall module linking this global haemodynamics to local fibre mechanics. In the Chamber module, one wall surrounds one cavity. In the TriSeg module, the two ventricles are encapsulated by three walls with strong mechanical interaction. The Bag module simulates the nonlinear constraining effect of the pericardium on the cardiac cavities together. Concerning blood circulation, the Valve module simulates the flow through the cardiac valves using Bernoulli’s principle [2]. The Tube0D module describes the nonlinear pressure-area relation of large blood vessels and ArtVen describes the flow through the microcirculation [2]. The PFC module is a negative feedback control system that enables homeostatic regulation of mean arterial pressure and systemic flow. This set of modules constitutes the set of key modules for building the model as detailed by Walmsley *et al*. [4].

This manuscript focusses on the accuracy of the closed-loop model. Verification of the individual modules and numerical integration methods demonstrating accurate implementation of these modules and methods can be found in the electronic supplementary material.

### (a) Closed-loop CircAdapt model

**Figure 1.**
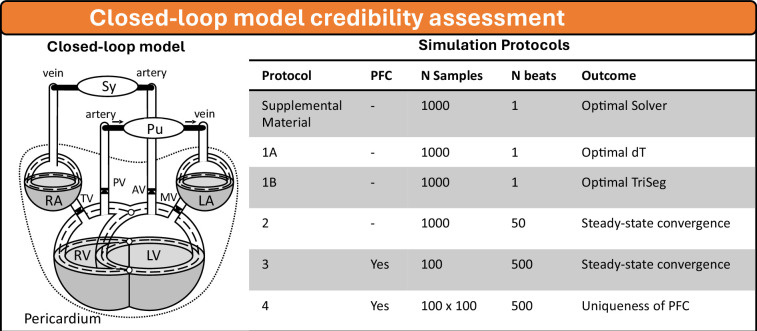
Overview of the closed-loop model (left) and the simulation protocols (right) utilized in this study. The closed-loop model is based on [4]. Sy: systemic resistance; Pu: pulmonary resistance; TV: tricuspic valve; PV: pulmonary valve; MV: mitral valve; AV: aortic valve; RA: right atrium; LA: left atrium; LV: left ventricle; RV: right ventricle;

We utilize the previously introduced closed-loop model [4], illustrated in figure 1. This model incorporates all the modules described in the benchmark set-ups (electronic supplementary material). In this model, the Chamber module and TriSeg module simulate global pump mechanics in the atria and ventricles, respectively. Both modules deploy the Wall module, which uses the one-fibre model to link local mechanics simulated with the Patch module to global pump mechanics. The Bag module simulates the effect of the pericardium and the surrounding tissues as an additional external pressure. The pulmonary and systemic circulations are modelled using two Tube0D cavities that simulate pressure–-volume relationships and an ArtVen module that simulates flow through the capillaries.

The numerical accuracy of this model is tested by varying 25 parameters representing contractility, stiffness, moment of activation, wall volume, wall reference area, valve insufficiency, heart rate, mean arterial pressure and cardiac output (table 1). These parameters cover a wide variety of relevant (patho-)physiological tissue substrates. These samples are used in four different protocols: (i) in the single-beat protocol, the numerical accuracy is investigated as a function of the numerical integration technique (electronic supplementary material), step size and TriSeg tension threshold; (ii) in the multi-beat protocol, propagation of accuracy in a steady state is determined without homeostatic PFC; (iii) in the PFC protocol, accuracy in steady state is determined with homeostatic PFC; (iv) in the PFC multi-start protocol, the uniqueness of the steady state with homeostatic PFC is quantified.

**Table 1.** The set of 25 model parameters with uniform sample ranges was used for the closed-loop accuracy analysis. The parameters A_leak and A_open each are applied in 25% of the samples. LVfw: LV free wall; RVfw: right ventricular (RV) free wall; IVS: inter ventricular septum; MV: mitral valve; AV: aortic valve.

model parameter	description	location	lower bound	upper bound	unit
**patch.Sf_Act**	contractility	LVfw	60	120	kPa
		IVS	60	120	kPa
		RVfw	60	120	kPa
**patch.k1**	passive stiffness exponent	LVfw	5	15	—
		IVS	5	15	—
		RVfw	5	15	—
**patch.Sf_pas**	passive stiffness constant	LVfw	100	2500	kPa
		IVS	100	2500	kPa
		RVfw	100	2500	kPa
**patch.dt**	activation delay of a patch	LVfw	0	50	ms
		IVS	0	50	ms
		RVfw	0	50	ms
**patch.V_wall**	wall volume	LVfw	80	120	%
		IVS	80	120	%
		RVfw	80	120	%
**patch.Am_ref**	wall reference area	LVfw	80	120	%
		IVS	80	120	%
		RVfw	80	120	%
**valve.A_leak**	leaking valve area (valve regurgitation)	MV	$10^{-10}$	75%: $10^{-10}$ 25%: 25	%
		AV	$10^{-10}$	75%: $10^{-10}$ 25%: 25	%
**valve.A_open**	open valve area (valve stenosis)	MV	75%: 100 25%: 75	100	%
		AV	75%: 100 25%: 75	100	%
**t_cycle**	cycle time	—	0.5	1.5	S
**PFC.p0^a^**	mean systemic arterial pressure	—	73	110	mm Hg
**PFC.q0^a^**	venous return	—	4	6	L min^−1^

Units expressed in % are relative to their reference values.

^a^ Parameters only applicable with PFC activated.

### (b) Single-beat protocol

A total of 1000 samples are drawn from the uniform distribution with ranges listed in table 1. Each simulation starts with the same state variables obtained from the steady-state reference. Simulations run for a single heartbeat using the integration methods listed in the electronic supplemental material. The simulations are tested with timesteps of $\Delta t=10^{-3},\;10^{-2},10^{-1},1,\;2,\;5,\;and\;\;10\;ms$. In addition, we test the TriSeg tension balance threshold at *e* = 10^–6^, 10^–5^, 10^–4^, 10^–3^, 10^–2^ and 10^–1^ N m^–1^. In these simulations, we examine the conservation of energy, the tension balance in TriSeg and the overall computation time. In addition, we analyse the mean absolute error in the left ventricular (LV) volume and LV pressure time signals. We assume the average signal obtained using various integration methods at the smallest timestep as the true value. All other calculated signals for each sample are compared to this assumed true value. The default numerical settings are selected to optimize computation time while maintaining a clinically relevant numerical error, defined as 0.1 ml for LV volume and 0.1 mm Hg for pressures.

### (c) Multi-beat protocol

To test convergence errors in haemodynamic steady state, we simulate 100 heartbeats for each of the 1000 samples drawn before. The assumed true steady state is obtained after simulating 100 heartbeats with the optimum integration method derived from the single-beat response with the smallest tested integration timesteps and tension balance threshold. Steady state is reached when


(2.1) $$\frac{n_{valve}}{\sum {\bar{q}}_{v,b}}\sqrt{\frac{\;\sum {\left({\bar{q}}_{v,b}-{\bar{q}}_{v,b-1}\;\right)}^{2}}{n_{valve}}}<e_{steady},$$

where ${\bar{q}}_{v,b}$ and ${\bar{q}}_{v,b-1}$ are the mean flow through valve v in beat b and the previous beat b-1, $n_{valve}=6$ is the number of valves in the circulation including the two venous-atria connections and $e_{steady}$ is the dimensionless PFC threshold determining steady state.

### (d) PFC protocol

To test convergence errors in a haemodynamic steady state with PFC, 250 heartbeats were simulated for 1000 samples drawn from the uniform distribution described above. The assumed true steady-state condition is obtained after simulating 250 heartbeats with the optimum integration method derived from the single-beat response with the smallest tested integration timesteps and tension balance threshold. In the CircAdapt framework, the PFC module is implemented to adapt effective blood volume and peripheral resistance to match the mean arterial pressure $p_{0,pfc}$ and venous return $q_{0,pfc}$ given as input parameters [2]. The sensing variable $s_{p}$ senses the mean arterial pressure $\bar{p}$ and the sensing variable $s_{q}$ senses the venous return $\bar{q}$:

(2.2) $$s_{p}={\left(\frac{\bar{p}}{p_{0,pfc}}\right)}^{f}and\;s_{q}={\left(\frac{\bar{q}}{q_{0,pfc}}\right)}^{f},$$

where f is a scaling factor. Both sensing variables equal 1 the in case of homeostatic steady state. The sensing variables control the effective blood volume by changing flow through the ArtVen objects such that $q_{prox}\neq q_{dist}$:

(2.3) $$q_{prox}=q_{artven}\cdot s_{p}\;\;and\;\;q_{dist}=q_{artven}\;/\;s_{p}.$$

The peripheral resistance $p_{0,artven}$ is iteratively updated after each heartbeat by


(2.4) $$p_{0,artven,i+1}=p_{0,artven,i}\cdot s_{q}/s_{p}.$$

The system reaches a homeostatic steady state, characterized by stationary waveforms, when


(2.5) $${\left(\frac{{\bar{q}}_{VR,b}}{q_{0}}-1\right)}^{2}+\frac{n_{valve}}{\sum {\bar{q}}_{v,b}}\sqrt{\frac{\sum {\left({\bar{q}}_{v,b}-{\bar{q}}_{v,b-1}\right)}^{2}}{n_{valve}}}<e_{steady},$$

where ${\bar{q}}_{v,b}$ and ${\bar{q}}_{v,b-1}$ are the mean flow through valve *v* in beat b and the previous beat b-1, $n_{valve}=6$ is the number of valves in the circulation, ${\bar{q}}_{VR,b}$ is the mean venous return, $q_{0,pfc}$ is the target venous return and $e_{steady}$ is the dimensionless PFC threshold determining steady state. Simulations were excluded if the mean left atrial pressure exceeded 50 mm Hg. The maximum left atrial pressure during exercise in healthy individuals is approximately 35 mm Hg [18]; however, we deliberately set our threshold at 50 mm Hg to include conditions that extend beyond the normal physiological range, such as those observed in patients with heart failure or severe haemodynamic disturbances. This approach allows us to better understand the model’s behaviour under pathological conditions and ensures that our analysis spans a wide spectrum of clinical scenarios. In addition, by including simulations with elevated pressures, we can identify the model’s limitations and evaluate its robustness in predicting outcomes under extreme conditions, which is critical for future applications in clinical decision-making.

### (e) PFC multi-start protocol

To validate the uniqueness of the steady-state simulation, we perform a multi-start simulation protocol. In this protocol, we use the same samples as in the previous protocol and simulate 100 heartbeats for each simulation to converge to a steady state. To reduce computational costs, only 100 samples were included. State variables from all successful simulations are used as a starting point for a multi-start analysis, meaning that each sample runs with 100 different starting points resulting in a total of 100 × 100 = 10 000 simulations. Convergence error for cardiac indices is calculated as the standard deviation of relevant cardiac indices in the last simulated heartbeat.

### (f) Numerical implementation

In this study, we used the pyCircAdapt version 2409 which is a Python 3 wrapper for the CircAdapt library written in C++. This software is available on http://framework.circadapt.org. All other code is written in Python 3 and is made available on GitHub (https://github.com/CircAdapt/VanOsta2024NumericalCredibilityAssessment). All simulations and analyses were conducted sequentially on a Windows laptop (Intel Core i7-11850H).

## 3. Results

### (a) Single-beat protocol

A total of 1000 simulations were run for a single heartbeat with each solver setting. Simulations using the fourth-order Adam-Bashforth, as well as all those with a 10 ms integration timestep, proved to be numerically unstable. At the integration timestep of $10^{-3}\;ms$ and TriSeg tension balance threshold of $e_{triseg}=10^{-6}$, all Adams–Moulton and backward differential formula solutions show similar accuracy, with volume and pressure differences less than $2\times 10^{-7}\;ml$ and $3\times 10^{-7}mm\;Hg$, respectively (electronic supplementary material, fig. S2). In contrast, the LV volume and pressure signals from other integration methods exhibit greater differences, with deviations reaching up to $3\times 10^{-3}\;ml$ and $3\times 10^{-3}\;mm\;Hg$.

At a step size of 1 ms, the Adams–Moulton and backward differential formula are the two most accurate methods. The second-, third- and fourth-order Adams–Moulton methods have an error of 0.020, 0.020 and 0.020 ml, respectively, in LV volume and 0.011, 0.011 and 0.011 mm Hg, respectively, in LV pressure. The simulations integrated with the backward differential method with second-, third- and fourth-order have an error of 0.020, 0.020 and 0.020 ml, respectively, in LV volume and 0.012, 0.011 and 0.011 mm Hg, respectively, in LV pressure. At a step size of 2 ms, the second-, third- and fourth-order Adams–Moulton methods have an error of 0.040, 0.041 and 0.041 ml, respectively, in LV volume and 0.027, 0.027 and 0.027 mm Hg, respectively, in LV pressure. The backward differential methods with second-, third- and fourth-order have an error of 0.040, 0.042 and 0.041 ml, respectively, in LV volume and 0.030, 0.028 and 0.027 mm Hg, respectively, in LV pressure. The accuracy of the integration methods other than Adams–Moulton and backward differential method was worse compared to these two methods.

**Figure 2.**
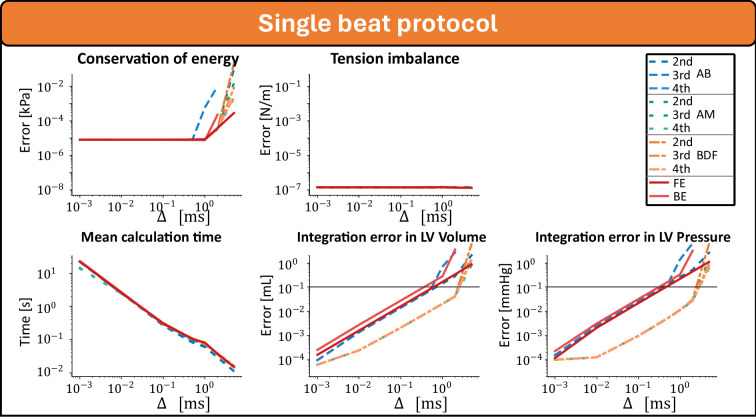
Integration error of the single-beat response as a function of the integration timestep. The true state is assumed to be the average of all integration methods at $\Delta t=10^{-2}\;ms$. Only mean values are displayed for readibility.

Figure 2 shows the integration error related to different integration step sizes for all seven methods with a TriSeg tension balance accuracy of $e_{triseg}=10^{-6}$. The error in energy balance, as well as the errors in the evaluated output volume and pressure signals decrease with reduced integration step size. The tension balance was independent of the step size. The integration error of the second-order Adams–Moulton had the overall lowest integration error compared with the assumed true state but was close to the integration error of the other orders and the backward differential formula.

**Figure 3.**
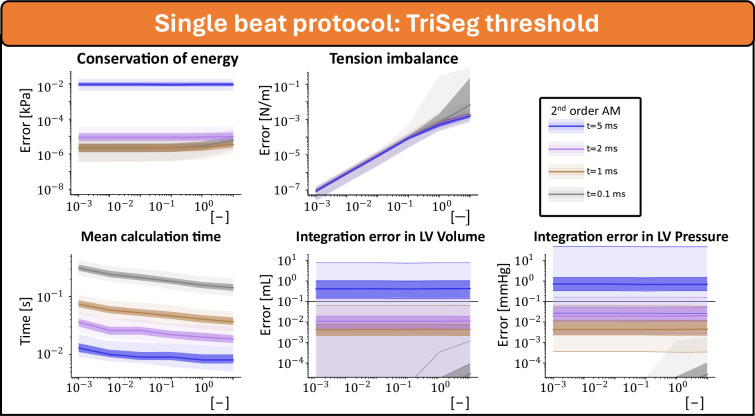
Integration error of the single-beat response as a function of the TriSeg tension balance. Simulations are conducted using the Adams–Moulton method with Δt values of $10^{-2}$, 1, 2 and 5 ms. The true state is assumed to be the result from the Adams-Moulton method with $\Delta t=10^{-2}\;ms$.

Figure 3 shows the integration error related to different TriSeg tension balance accuracies for the second-order Adams–Moulton method with step sizes of $10^{-1}$, 1, 2 and 5 ms. For all four settings, the tension balance converged towards zero with a lower threshold for the TriSeg force balance. Calculation time was mostly determined by the integration timestep, but also slightly reduced by a higher threshold for the TriSeg force balance. Up to an integration timestep of 2 ms, clinically relevant accuracy was obtained. This accuracy was independent of accuracy in force balance, suggesting that the step size dominates the accuracy.

### (b) Multi-beat protocol

**Figure 4.**
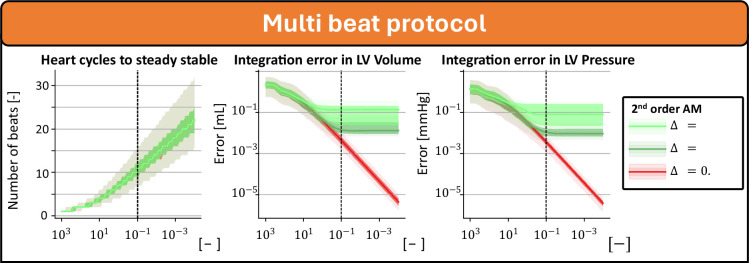
Convergence and integration error of steady-state simulations in the multi-beat protocol. The assumed steady state is derived from the last beat of simulations integrated with the Adams–Moulton method at Δt=0.1ms over 100 beats to reach steady state.

Figure 4 shows the steady-state results of 1000 simulations without homeostatic PFC. All simulations were numerically stable and converged to a steady state at the lowest tested steady-state threshold after 100 beats, except those with an integration timestep of 5 ms. By comparing the pressure and volume traces with the assumed true steady-state value, convergence error in the simulations integrated with a timestep of 1 ms and 2 ms did not decrease after the steady-state threshold of $e_{steady}=10^{-1}$ was reached. Using these settings, a steady state was found after a calculation time of 349 ms (90% CI 235–645 ms) and 214 ms (90% CI 137–323 ms) for the method with integration timestep 1 and 2 ms, respectively.

### (c) PFC protocol

**Figure 5.**
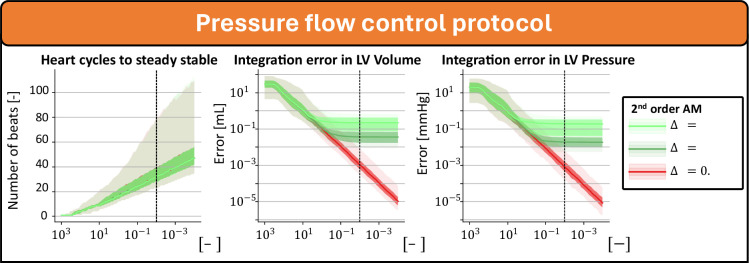
Convergence and integration error of steady-state simulations with PFC. The assumed steady state is derived from the last beat of simulations integrated with the Adams–Moulton method at Δt=0.1ms over 250 beats to reach steady state.

Of the 1000 samples, 818 simulations were numerically stable, and they all reached a steady state (figure 5). In all numerically unstable simulations, the mean left atrial pressure in the last stable heartbeat was greater than 50 mm Hg, suggesting these simulations are beyond physiological ranges.

At a steady-state threshold of $e_{steady}=10^{-2}$, convergence errors of the evaluated indices were close to the integration errors as the error did not further reduce by decreasing the threshold. At this threshold, the calculation time was 1290 ms (90% CI 711–3095 ms) and 642 ms (90% CI 352−1588 ms).

### (d) PFC multi-start protocol

The state variables of 100 simulations were used as starting point to run 100 new simulations for each starting point, resulting in a total of 10 000 simulations from which 9939 (99%) simulations were numerically stable. For the remaining 61 unstable simulations, the steady state was known from other starting points, and in these known steady states, the left heart filling pressure was greater than 50 mm Hg, meaning these 61 simulations were not plausible. The median of the mean absolute error relative to the mean signal was $7.1\times 10^{-6}\;ml$ (95% CI: $1.7\times 10^{-10}$–$4.5\times 10^{-3}\;ml$) for the LV volume signal and $1.2\times 10^{-6}\;mm\;Hg$ (95% CI: $1.4\times 10^{-9}$–$2.9\times 10^{-2}\;mm\;Hg$) for the LV pressure signal.

## 4. Discussion

In this study, we demonstrated that achieving a clinically accurate steady state very much depends on the simulation conditions. In our use case, it required 7–15 heartbeats when no regulation is included. When homeostatic control mechanisms are used to control mean peripheral resistance and effective blood volume, more than double the above-mentioned number of heartbeats was needed in the simulations to reach an accurate steady stable simulation. Numerical inaccuracies were optimally balanced with computational cost when employing the Adams–Moulton integration method with a timestep size of 1 ms, which provided clinically accurate results. While it is intuitive that the number of simulated heartbeats influences the accuracy of the steady state, this relationship has been underexplored in the field of cardiovascular system models. Understanding this relationship becomes increasingly important as these models advance toward clinical applications.

### (a) Verification of the CircAdapt framework

To distinguish between steady-state convergence errors and other numerical errors, we verified the numerical implementation of all modules and validated a general-purpose closed-loop model representing the healthy adult heart and circulation. To do so, we analyzed the LV pressure and volume traces, aiming for clinically relevant accuracy within 0.1 mm Hg and 0.1 ml, respectively. Based on the accuracy in LV volume and pressure signals, we determined that using the Adams–Moulton method with a 1 ms integration timestep, a tension threshold of $e_{triseg}=10^{-1}$ and a steady-state threshold of $e_{steady}=10^{-3}$ provided sufficient accuracy. These settings offered a good balance between precision and computational speed. As a result, the default model set-ups in the CircAdapt framework employ this numerical configuration. All modules, verification steps and the default model set-up are thoroughly documented and openly available (framework.circadapt.org).

The verification of computational models is receiving more attention, particularly as translational research becomes more integrated with clinical applications [19]. This is underscored by frameworks for credibility assessment provided by the US Food and Drug Administration and American Society of Mechanical Engineers [9,10]. Consequently, more research software is undergoing verification, often through comparison with analytical solution [20,21] or by utilizing the method of manufactured solutions [22]. This study focusses on verifying individual modules and quantification of numerical uncertainties in a general-purpose model, providing a blueprint for verification of new to-be-added modules to the CircAdapt framework and for addressing numerical uncertainties in specific projects. The analytical module verification presented in this paper is included in the unit tests as part of the pyCircAdapt package to ensure correct implementation.

With the introduction of the MultiPatch module [4]—the Wall module in the CircAdapt framework—the one-fibre model as reported by Arts *et al*. [6] has been slightly reformulated. The original one-fibre model described fibre stress-to-cavity pressure ratio in relation to the cavity and wall volume [6]. Over time, the one-fibre model has been restructured to describe tension at the mid-wall, offering a symmetric representation of wall mechanics, which is crucial for the TriSeg module [5]. In addition, the model has been linearized to facilitate wall segmentation [4]. Despite these modifications, our results in figures 2 and 3 demonstrate that the model still adheres to the conservation of energy, a fundamental principle of the one-fibre model [6]. Although linearization introduces a small discrepancy (electronic supplementary material), this effect is probably counterbalanced by other mechanisms within the fully coupled closed-loop model. With new modules being developed [23–25] and the application of the CircAdapt framework in clinical scenarios becoming more prevalent [26–28], this study illuminates the sources of numerical uncertainty, enabling better optimization between accuracy and computational efficiency.

### (b) Accuracy of closed-loop simulations

As the steady-state simulation is often used in research, the total computational cost of a steady-state simulation is the cost of simulating a single heartbeat multiplied by the number of heartbeats required to reach this steady state. This steady state is reached through redistribution of volume over the system but can also include homeostatic PFC to mimic various physiological processes [8]. While often steady state is assumed after a fixed number of heartbeats, our results demonstrate this is not the case.

In the closed-loop model set-up investigated in this study, multi-step implicit methods demonstrated the highest accuracy, particularly owing to their stability in stiff problems. These methods outperformed others in benchmarks A and C (electronic supplementary material), where the ordinary differential equations were similar while showing no significant difference in accuracy for benchmarks B and D (electronic supplementary material). The closed-loop model was fast, numerically stable and accurate, over a broad parameter range representing physiological and pathophysiological cardiovascular substrates. When extending the model with additional modules, such as the one-dimensional wave propagation model of vascular mechanics and haemodynamics [29], or by coupling the novel calcium-contraction model [23] to cardiac electrophysiology models operating on smaller timescales [30], other techniques are available to improve stability and accuracy and to reduce computational time. For example, in models of cardiac electrophysiology, the Rush–Larsen scheme solves the gating variables of ion channels semi-analytically, which improves stability in the simulation [31]. Variable integration methods such as the Adams–Bashforth–Moulton method, as used in previous implementations of the CircAdapt model [4], could further reduce computation time without losing accuracy. In addition, multi-rate integration methods could further reduce computational costs [32]. Future studies will focus on the implementation of these additional modules, including appropriate verification steps, unit tests and documentation.

Cardiovascular mechanics models typically involve more complex partial differential equations problems where analytical solutions are often unavailable, making verification challenging. Verification of these problems is often limited to cross-validation between different model implementations [33–35]. Recent guidelines focus on validation and uncertainty quantification [36]. Despite these efforts, convergence errors, in steady-state conditions remain, to the best of our knowledge, poorly studied. This highlights a gap in the current credibility assessment. In complex mathematical applications, such as digital twins [26–28] or virtual cohorts [37] in which steady-state results are compared with target values, this uncertainty regarding the steady-state condition is fully ignored, even though the number of beats needed for steady state is a main contributor to computational cost. Various approaches have been introduced to reduce computational cost, including reduced-complexity models [1] and Gaussian emulators of complex models [38]. While some studies omit steady-state criteria [26–29] or use a predetermined number of heartbeats to achieve a steady state [3,38–40], our analysis suggests that the number of simulated beats to reach a steady state can be adapted to the specific protocol, while reducing the computational cost and ensuring an accurate steady state across a wide parameter range.

## 5. Conclusion

We have demonstrated that achieving a clinically accurate steady state requires calculating the appropriate number of heartbeats, which varies across simulations depending on initial state variables and parameterization. Accounting for this variability ensures an efficient balance between computational cost and steady-state accuracy. Notably, the required number of heartbeats increases when homeostatic parameter adaptation is included. Our study highlights the balance between accuracy and computational efficiency.

## Data Availability

Supplementary material is available online [41].
